# Effectiveness, safety and tolerability of a complex homeopathic medicinal product in the prevention of recurrent acute upper respiratory tract infections in children: a multicenter, open, comparative, randomized, controlled clinical trial

**DOI:** 10.1186/s40248-016-0056-1

**Published:** 2016-05-16

**Authors:** Miek C. Jong, Stephen L. Buskin, Lydia Ilyenko, Irina Kholodova, Julia Burkart, Stephan Weber, Thomas Keller, Petra Klement

**Affiliations:** Department Nutrition & Health, Louis Bolk Institute, Hoofdstraat 24, 3972 LA Driebergen, The Netherlands; Department of Health Sciences, Mid Sweden University, Sundsvall, Sweden; National Information and Knowledge Center on Integrative Medicine (NIKIM), Amsterdam, The Netherlands; International Health Center of the Hague, The Hague, The Netherlands; Russian State Medical University, Moscow, Russia; Deutsche Homöopathie-Union, DHU-Arzneimittel GmbH & Co. KG, Karlsruhe, Germany; Acomed statistik, Leipzig, Germany

**Keywords:** Homeopathy, Complex homeopathic medicinal product, Immunokind®, Children, Upper respiratory tract infections, Prevention, Antibiotics, Randomized controlled clinical trial

## Abstract

**Background:**

The present study was initiated to investigate the effectiveness, safety and tolerability of complex homeopathic CalSuli-4-02 tablets on prevention of recurrent acute upper respiratory tract infections (URTIs) in children, in comparison to another complex homeopathic product.

**Methods:**

The study was designed as a prospective, multicenter, randomized, open, clinical trial with two parallel treatment groups at four outpatient pediatric clinics in Russia. Children aged ≤ 6 years with susceptibility to acute URTIs (≥ three occasions during the last 6 months) were randomized to receive either CalSuli-4-02 or a comparator homeopathic product (control group) for 3 weeks. Primary outcome was the frequency of acute URTIs after 3 and 6 months post-treatment follow-up. Secondary endpoints were changes in complaints and symptoms (total and individual scores), treatment satisfaction, antibiotic use, safety and tolerability.

**Results:**

The intention-to-treat analysis involved 200 children (CalSuli-4-02: *N* = 99, Control: *N* = 101). In both treatment groups, the median number of acute URTIs was one for 3 months and two, respectively, for the full 6 months post-treatment (Relative Risk: 0.86 (95 %-CI: 0.72–1.03), *p = 0.1099*). Seasons had no influence on the outcome. At the end of study, CalSuli-4-02 had overall higher odds of getting lower complaints severity total score (Odds ratio: 1.99 (95 %-CI: 1.31–3.02), *p = 0.0012*) and showing symptom improvement (Odds ratio: 1.93 (95 %-CI: 1.25–3.00), *p = 0.0033*). Specifically, the complaint “appetite disorder” and the symptom “child’s activities” significantly improved more in the CalSuli-4-02 group (*p = 0.0135* and *p = 0.0063*, respectively). Antibiotic use was decreased in both treatment groups at the study end. Overall assessment for satisfaction with and tolerability of treatment was higher with CalSuli-4-02. A low number of non-serious adverse drug reactions was reported (CalSuli-4-02: *N* = 4, Control: *N* = 1).

**Conclusions:**

Both complex homeopathic products led to a comparable reduction of URTIs. In the CalSuli-4-02 group, significantly less URTI-related complaints and symptoms and higher treatment satisfaction and tolerability were detected. The observation that the use of antibiotics was reduced upon treatment with the complex homeopathic medications, without the occurrence of complications, is interesting and warrants further investigations on the potential of CalSuli-4-02 as an antibiotic sparing option.

**Clinical trial registration number:**

Roszdravnadzor: Study No 164–563

## Background

Acute upper respiratory tract infections (URTIs) and acute ear, nose and throat (ENT)-diseases such as the common cold, otitis media, sinusitis, pharyngitis and tonsillitis frequently occur in young children [1, 2]. URTIs are often viral conditions that are self-limiting in nature. Standard care often involves symptom management with medications such as cough and cold preparations and nasal decongestants. Antibiotics are also frequently prescribed for URTIs, despite the fact that they are often not indicated as in most cases URTIs are viral in origin [3]. A recent pharmacoepidemiologic study demonstrated that homeopathic medications were the second most frequently used medications in Germany to treat URTIs in children [4]. Homeopathy was developed by the German physician and chemist Samuel Hahnemann and homeopathic medications are manufactured from herbal, mineral, animal, or other natural substances and diluted through concessive rounds of vigorous shaking [5, 6]. The dilution factors of the homeopathic medications are mostly described as 1/10 dilution ratio steps (D-series) or 1/100 dilution ratio steps (C-series) [5, 6]. Worldwide, many children are treated by homeopathy [7–9]. Either through self-management (bought over the counter) or prescription by homeopathic practitioners, children can be treated individually with single homeopathic medications, based on the totality of the symptom pattern that the child presents. Children may also use complex homeopathic medications, containing usually two to six active homeopathic substances, which are sold over the counter for self-limiting conditions [5, 6]. According to a review, prevalence rates for the use of homeopathy are the highest in Germany, UK and Canada and range altogether for all involved countries from 0.8 to 39 % for lifetime use and from 1 to 14.3 % for current use [8]. Of all pediatric illnesses, parents most frequently seek homeopathy to treat respiratory complaints in their children and the rationale for parents to choose homeopathy for their children is predominantly guided by advice and good experience of family and friends [10, 11].

Several studies have been performed suggesting that homeopathy may be effective in the treatment of URTIs in children. A study by Haidvogl et al. [12] demonstrated that overall, individual homeopathic treatment in children is just as effective in the treatment of acute URTIs compared to standard, conventional care; however onset of improvement within the first seven days after treatment was significantly faster upon homeopathic treatment. In two other studies, one in children with acute otitis media treated with individual homeopathic treatment [13] and one in children and adults with feverish URTIs receiving a complex homeopathic medicinal product as add-on to conventional treatment [14], initial symptomatic improvement was significantly faster upon homeopathic treatment compared to conventional treatment alone. A placebo-controlled study with complex homeopathic ear drops demonstrated that homeopathy was moderately effective in treating acute otitis media in children, but also more effective in the early period after diagnosis [15]. A systematic review concluded overall that homeopathic treatments may help decrease pain and lead to faster resolution of acute otitis media [16]. Besides possible effectiveness of homeopathy in the treatment of URTIs, previous studies have also reported promising effects of homeopathic treatment in the prevention of URTIs in children. Evaluation of thirty case series demonstrated that the average number of URTIs significantly decreased upon individualized homeopathic treatment compared to before the start of treatment [17]. In addition, a randomized study with a waiting list control group showed that children receiving individualized homeopathic treatment experience fewer days with URTIs and fewer symptoms from the URTIs than those in the control group [18]. All these studies indicate that homeopathic treatment, both individually prescribed homeopathic and complex homeopathic medications, may be beneficial in the treatment of URTIs in children but also in their prevention by empowering the child’s immune system along with decreasing the child’s susceptibility to catch infections.

The complex homeopathic medication, CalSuli-4-02 tablets (Immunokind®, Deutsche Homöopathie-Union, DHU-Arzneimittel GmbH & Co. KG), is sold over the counter in many European and non-European countries for increase resistance to recurring URTIs in infants and children of early age. Originally, it was developed in the Netherlands by homeopathic physicians and marketed since 1984. The present randomized, controlled clinical trial aimed to investigate the effectiveness, safety and tolerability of CalSuli-4-02 tablets in the prevention of recurrent acute URTIs in children. The study was directed to the effect of the complex homeopathic medication rather than to the homeopathic care. Since this clinical trial was initiated as to require marketing authorization in the Russian Federation (RF), the Russian regulatory authorities requested to compare the effectiveness and safety of CalSuli-4-02 tablets with a comparator homeopathic product, already marketed in the RF for prevention of URTIs.

## Methods

### Trial design

A prospective, multicenter, randomized, open-label, comparative, controlled clinical trial with two parallel groups was conducted in the RF, in accordance with the legislation of the RF and its national standards of Good Clinical Practice. The study protocol was approved by the independent Ethics Committee of the RF (Protocol No. 50, November 11, 2009), the local Ethics Committees of the four participating study centers and by the regulatory authorities of the Ministry of Health and Social Development of the RF (Protocol No. 563, December 28, 2009). On December 31, 2010, approval for a 1-year prolongation of the clinical study (Protocol No. 563) was obtained from the Ministry of Health and Social Development of the RF. The data of the present clinical trial were first analyzed in RF and submitted to the Ministry of Health and Social Development of the RF in December 2011. The data presented in this publication are based on a new analysis from 2014 to 2015, in line with International Conference on Harmonization (ICH) guidelines.

### Participants

Children were allowed to participate in the study if they met the following inclusion criteria: boy or girl, up to 6 years of age, three or more episodes of acute URTIs (diagnosis based on World Health Organization (WHO) International statistical Classification of Diseases and related health problems (ICD)-10 code J06.9 documented in the child’s medical record) in the last 6 months prior to the study start, and signed informed consent from parents for participation of their child in the study. Children were excluded from participation if they had an acute URTI or exacerbation of a chronic URTI upon starting the study, severe concomitant diseases (renal failure, heart anomalies, circulatory failure, cardiomyopathy, decompensated kidney and liver, immunosuppressive conditions, oncological diseases), known or suspected hypersensitivity to any component of the study medication, or in case of participation in other clinical studies or use of immune-modulatory medications or being vaccinated against influenza within the last 6 months before the start of the study. During the study period children were not allowed to use immune-modulatory medications and prophylactic medications for URTIs, or being vaccinated. The study took place at four outpatient pediatric clinics in the RF: the State Educational Institution for Additional Professional Education (Russian Medical Academy of Postgraduational Education) and the State Educational Institution for Higher Professional Education (Russian State Medical University) in Moscow, the State Educational Institution for Higher Professional Education (Smolensk State Medical Academy) in Smolensk, and the State Educational Institution for Higher Professional Education (Nizhegorodksaya State Medical Academy) in Nizhniy Novgorod. Pediatricians of all four participating clinics informed parents of children with frequent acute URTIs about the study and directed them to the study investigators, who consequently invited them to take part in the study, until the planned sample size was reached. The first child was included in the study on February 15, 2010 and the last child completed the study on September 7, 2011.

### Interventions

The intervention group was treated with the investigational product CalSuli-4-02 tablets for a period of 3 weeks with a dosage regimen of one tablet, three times a day. For children aged < 3 years the CalSuli-4-02 tablet was dissolved in 5 ml (1 teaspoon) water to facilitate intake. CalSuli-4-02 is a complex homeopathic medicinal product containing four active ingredients: Calcium carbonicum Hahnemanni D6, Calcium fluoratum D6, Calcium phosphoricum D6 and Sulfur jodatum D12. The control group was treated for a period of 3 weeks with a comparator complex homeopathic medicinal product that consisted of the following five active ingredients: Gentiana D1, Aconitum D6, Bryonia D6, Ferrum phosphoricum D12, and Acidum sarcolacticum D12. For children aged < 1 year, half of a tablet of the comparator product was dissolved in one teaspoon of water or breast milk and one drop of that solution was administered two times a day. For children aged 1–6 years, half a tablet was administered two times a day. In case of acute URTIs and other ENT diseases during the study period, the children’s symptoms were treated with antipyretics and/or other symptomatic treatments (e.g. antitussives, nasal decongestants, ear drops and throat gargle). If indicated, antibiotics were prescribed. The use of any kind of drug was documented in the child’s medical record, which was kept by the parents. Concomitant medication was not additionally included in trial documentation and monitoring.

A total of five study visits were scheduled and 200 children were planned to be randomly allocated either to the CalSuli-4-02 or control group. After the start of treatment, four follow-up (FU) Visits took place. The 1^st^ FU Visit was 3-5 days and the 2^nd^ FU Visit 3 weeks (21 days) after the start of study treatment. The 2^nd^ FU Visit was also the end of treatment period and the study medication was returned. The 3^rd^ FU Visit was 3 months post-treatment and the last Visit (Termination Visit), 6 months post-treatment. The individual duration for children in the study, therefore, added up to 201 days, including the 3 weeks (21 days) treatment and 6 months post-treatment period. Every time during the follow-up period when a child felt sick with acute complaints in the upper respiratory tract, the child consulted a pediatrician. The pediatrician assessed the clinical symptoms and the primary diagnosis according to classification of WHO ICD-10, which was documented in the child’s medical record. In case the clinical symptoms applied to an acute URTI, the WHO ICD-10 code J06.9 was documented and the case was counted as acute URTI for study purposes.

### Outcome

The objective of the study was to assess the effectiveness, safety and tolerability of CalSuli-4-02 compared to another complex homeopathic medicinal product in the prevention of acute URTIs in children. The primary outcome parameter was the frequency of acute URTIs assessed at the 3^rd^ FU Visit (1–3 months post-treatment) and at Termination Visit (4–6 months and full 6 months post-treatment) by means of documented URTIs (WHO ICD-10 code J06.9) in the child’s medical record within the respective time period. Secondary outcome parameters were changes in total scores and severity of individual complaints (fatigability, cough, nasal discharge (blocked/runny nose), appetite disorder, irritability – each of the above with a maximum of two points, and a maximum total score of ten points) and objective or symptoms examined by the investigator (fever, nasal discharge (rhinorrhea/mucopurulent), skin pallor, rales in lungs, restlessness for unknown reason, atopic dermatitis manifestations, child’s activities impairment – each of the above with a maximum score of two points, except atopic dermatitis: maximum four points and skin pallor: maximum one point, and a maximum total score of 15 points). Complaints and objectives were evaluated and scored at each visit by the investigator either according to children’s/parents’ self-report or according to the child’s examination results. The total scores were calculated by the investigator based on the single answers. Other secondary outcome was treatment satisfaction assessment by children/parents using the 5-point verbal rating Integrative Medicine Patient Satisfaction Scale (IMPSS, very satisfied, satisfied, neutral, dissatisfied, very dissatisfied [19]) at the 2^nd^, 3^rd^ FU and Termination Visit. Furthermore, the use of antibiotics with respect to treatment of URTIs and other ENT diseases such as sinusitis, otitis media and bronchitis was assessed as always (used in all occasions that there was an URTI/ENT), sometimes (estimated by the physician), seldom (estimated by the physician) or never (in none of the occasions antibiotics were used), based on the documentation in the child’s medical record at baseline, 3^rd^ FU and Termination Visit. Secondary outcome regarding safety of study treatment was assessed by systematic review of the incidence of adverse events (AEs) and adverse drug reactions (ADRs). Tolerability of treatment was evaluated by investigator’s and children’s/parents’ assessment using a 4-point verbal rating scale (excellent, good, satisfactory, poor) at the 1^st^, 2^nd^ FU and Termination Visit.

### Sample size

The study has an explorative character and, therefore, a formal sample size calculation was not necessarily required. However, the planned number of children had been justified as follows: a treatment related difference in acute URTI occurrence of one event was assumed as clinical meaningful difference and simulations (Poisson regression modelling) with the sample size of 100 children in each treatment group were performed to obtain the power of the study. It was calculated that if the mean number of acute URTIs 1 year prior to study start, is four or less, and a difference of one event is taken into account, the study would have more than 82.8 % power to detect the effect (0.05 % alpha level).

### Randomization

The randomization list was generated by the Laboratory of Biostatistics State Research Center for Preventative Medicine (Moscow, RF) and the random block size was four. At each center, children were assigned a study number in ascending order based on entry in the study. For each study number, the investigator received a sealed envelope containing the name of the investigational product to be given to the child according to the randomization list. The envelope was opened after the children’s parents had provided signed informed consent.

### Statistical methods

All effectiveness analyses were based on the intention-to-treat (ITT) analysis principle. The ITT population included those children who were randomized, had received at least one dose of study medication and had at least one post-baseline response measurement. Safety analysis included all randomized children, who received at least one dose of study medication. The homogeneity of the two treatment groups was assessed by regarding possible clinical relevance of group specific differences of (demographic) data at baseline. Primary outcome data were presented by descriptive statistics. As primary analysis method, post-treatment frequencies of acute URTIs were investigated by Poisson regression modelling. Treatment related difference in response was reported as relative risk (RR) presented with their 95 %-confidence interval (CI) and related *p*-values. Secondary variables were presented by descriptive statistics. To test for treatment related differences ordinal logistic regression using the proportional odds model (POM) and repeated measures of covariance analysis (ANCOVA) were applied for change of complaints and symptoms severity total score. For the other secondary variables chi-square (*χ*^2^)-tests were performed to test for treatment related differences. A rejection-criterion of 0.05 was set for all statistical tests. If tests allowed, the statistics were two-tailed.

## Results

### Study population

**Fig. 1:**
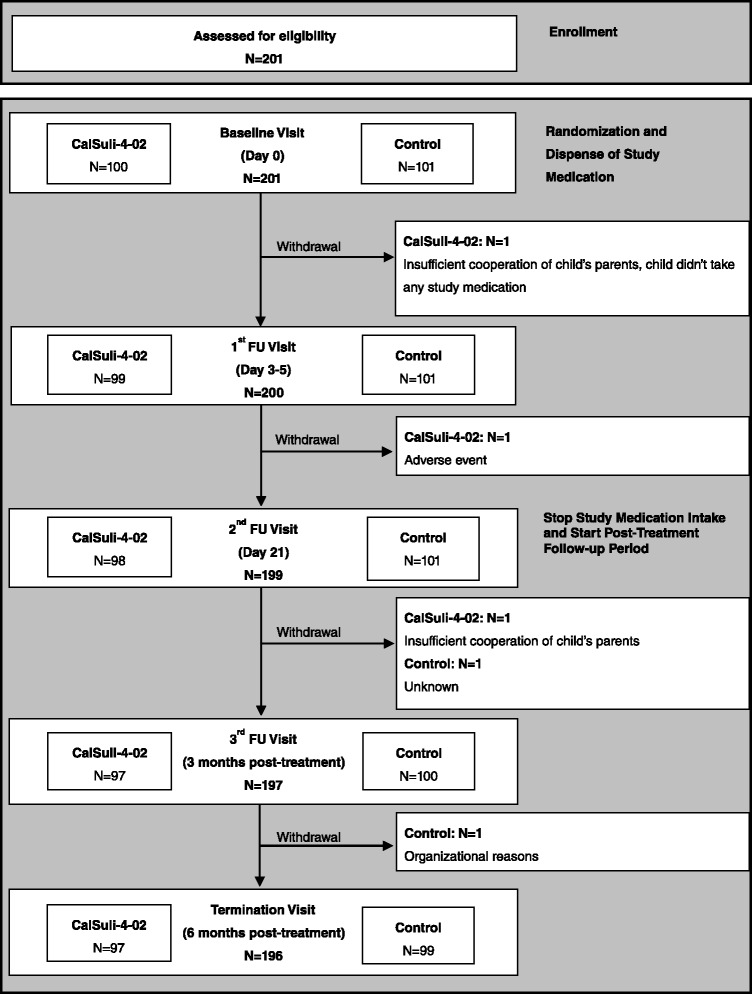
Flow diagram of children in the study. ITT population consisted of 200 children, since one child in the CalSuli-4-02 group didn’t take study medication. *FU* follow-up

Figure 1 depicts a flow diagram of the 201 children (CalSuli-4-02: *N* = 100, Control: *N* = 101) enrolled in the study. The ITT analysis consisted of 200 children (CalSuli-4-02: *N* = 99, Control: *N* = 101), since one child in the CalSuli-4-02 group didn’t take study medication. With respect to demographic and clinical characteristics, no clinically relevant differences were observed between the two treatment groups (Table 1). The number of acute URTIs in the 12 months period prior to study start was 6.0 (median) in both treatment groups (Table 1), whereof at least three acute URTIs per child were in the last 6 months prior to study start.

**Table 1:** Demographic and clinical characteristics

Characteristics	Statistics	CalSuli-4-02 group *N* = 99	Control group *N* = 101
Age (months)	Mean ± SD	34.2 ± 20.1	35.8 ± 20.0
	Median	34.0	34.0
	P25 %–P75 %	13.0–50.0	18.0–50.0
	Min–Max	5.0–72.0	6.0–73.0
Gender			
Boys / Girls	N	52 / 47	53 / 48
Number of acute URTIs (12 months prior to study start)	Mean ± SD	6.8 ± 2.1	6.7 ± 1.9
	Median	6.0	6.0
	P25 %–P75 %	6.0–8.0	6.0–7.6
	Min–Max	3.0–12.0	3.3–12.0

*SD* standard deviation, *URTIs* upper respiratory tract infections, ITT intention-to-treat analysis

### Primary outcome parameter

**Fig. 2:**
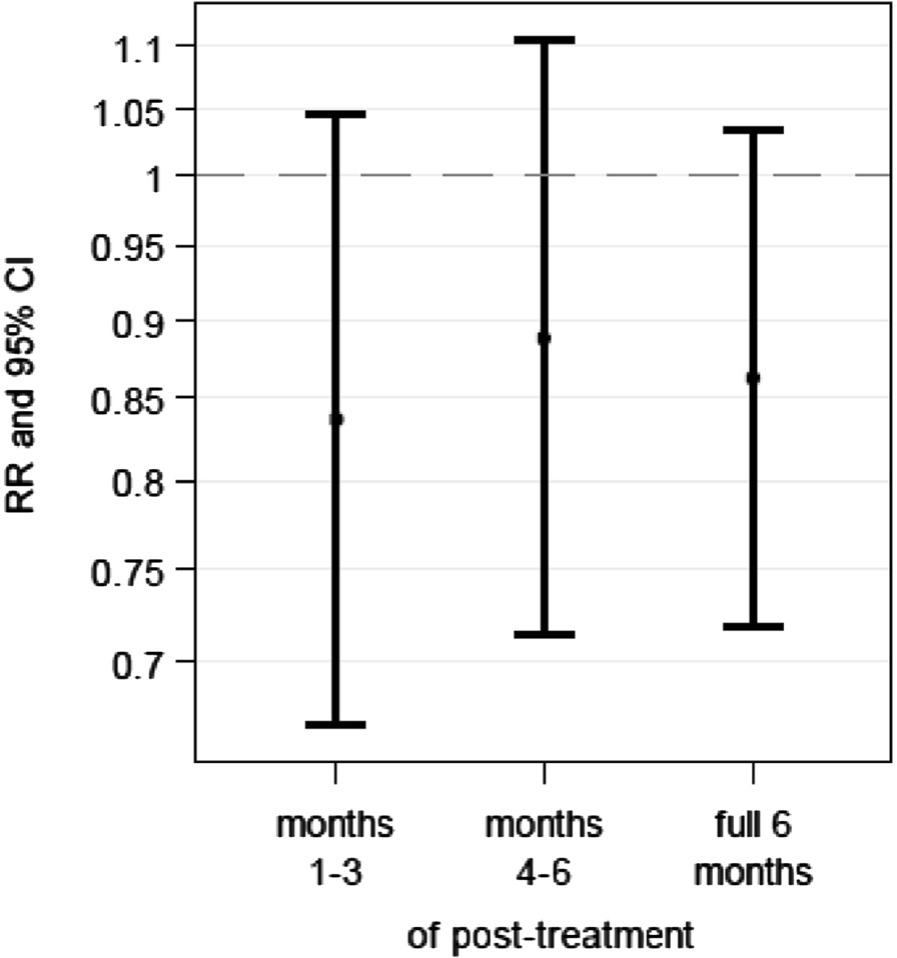
Primary outcome: Relative risks of experiencing acute URTIs. Line plots for relative risks in least square means (i.e. the estimated risk of experiencing an URTI) depicted as the differences between CalSuli-4-02 and control. *CI* confidence interval, intention-to-treat analysis

In both treatment groups, the median number of acute URTIs was two for the full 6 months of post-treatment. These were subdivided into one each for the months 1–3 as well as 4–6 of post-treatment (Table 2). Baseline adjusted Poisson regression modelling demonstrated that the risk of experiencing acute URTIs within the full 6 months of post-treatment was comparable in both treatment groups (RR in Table 2 and Fig. 2). The observed effect of RR = 0.86 (95 %-CI: 0.72–1.03) indicated a slightly lower risk of experiencing acute URTIs in the CalSuli-4-02 group compared to the control group. However, the effect was not statistically significant (*p = 0.1099; ITT*). Comparable results were obtained for the post-treatment periods of 1–3 and 4–6 months (Table 2 and Fig. 2). Further baseline-adjusted Poisson regression model analysis demonstrated that the covariates seasonal influence and prior antibiotics use had no significant influence on the frequency of acute URTIs (results not shown).

**Table 2:** Descriptive statistics and relative risk of treatment related effects on acute URTI frequencies

Post-treatment period	Treatment group	Number of acute URTIs experienced			Relative risk (RR)	
		Mean ± SD	Median (P25 %, P75 %)	Min-Max	Estimate (Lower -Upper 95 %-CI)	*p*
Months 1–3 of post-treatment	CalSuli-4-02	1.1 ± 1.0	1.0 (0.0, 2.0)	0.0–5.0	0.84 (0.67–1.05)	* 0.1185*
	Control	1.3 ± 0.9	1.0 (1.0, 2.0)	0.0–4.0		
Months 4–6 of post-treatment	CalSuli-4-02	1.1 ± 0.9	1.0 (0.0, 2.0)	0.0–4.0	0.89 (0.71–1.10)	* 0.2857*
	Control	1.2 ± 0.9	1.0 (0.0, 2.0)	0.0–3.0		
Full 6 months of post-treatment	CalSuli-4-02	2.2 ± 1.6	2.0 (1.0, 3.0)	0.0–7.0	0.86 (0.72–1.03)	* 0.1099*
	Control	2.5 ± 1.4	2.0 (1.0, 4.0)	0.0–6.0		

*CI* confidence interval, *RR* relative risk (i.e. the estimated risk of experiencing an event in children treated with CalSuli-4-02 divided by the estimated risk of experiencing an event in children treated with the comparator homeopathic product as obtained from Poisson regression model), *SD* standard deviation, *URTIs* upper respiratory tract infections, ITT intention-to-treat analysis

### Secondary outcome parameter: complaints and symptoms severity total score

The complaints and symptoms severity total score were already low in both treatment groups at baseline (median 3.0 points, out of 10.0 points and 15.0 points maximum, respectively), but further decreased until Termination Visit (Table 3). Within both treatment groups the decrease from baseline to the respective Visits was significant (ANCOVA: *p < 0.05* for all post-baseline Visits*; ITT*). In a baseline adjusted POM, children receiving CalSuli-4-02 had overall higher odds of showing improvement by means of getting lower complaints severity total score (Odds ratio (OR): 1.99 (95 %-CI: 1.31–3.02), *p = 0.0012; ITT*) and lower symptoms severity total score (OR: 1.93 (95 %-CI: 1.25–3.00), *p = 0.0033, ITT*) as those receiving the comparator homeopathic product.

**Table 3:** Complaints and symptoms severity total score

Outcome measure	Visit	Treatment group	Total score			Overall odds ratio (OR)	
			Mean ± SD	Median (P25 %, P75 %)	Min-Max	Estimate (Lower - Upper 95 %-CI)	*p*
Complaints	Baseline (day 0)	CalSuli-4-02	3.3 ± 2.0	3.0 (2.0, 5.0)	0.0–8.0	1.99 (1.31–3.02)	*0.0012*
		Control	3.3 ± 2.0	3.0 (2.0, 4.0)	0.0–7.0		
	2^nd^ FU (day 21)	CalSuli-4-02	1.1 ± 1.3	1.0 (0.0, 2.0)	0.0–6.0		
		Control	1.6 ± 1.5	1.0 (0.0, 3.0)	0.0–6.0		
	3^rd^ FU (3 months p-t)	CalSuli-4-02	0.9 ± 1.1	0.0 (0.0, 1.0)	0.0–5.0		
		Control	1.4 ± 1.5	1.0 (0.0, 3.0)	0.0–5.0		
	Termination (6 months p-t)	CalSuli-4-02	0.6 ± 1.1	0.0 (0.0, 1.0)	0.0–6.0		
		Control	0.9 ± 1.1	0.0 (0.0, 2.0)	0.0–4.0		
Symptoms	Baseline (day 0)	CalSuli-4-02	2.6 ± 1.9	3.0 (1.0, 4.0)	0.0–9.0	1.93 (1.25–3.00)	*0.0033*
		Control	2.6 ± 2.0	3.0 (1.0, 4.0)	0.0–8.0		
	2^nd^ FU (day 21)	CalSuli-4-02	1.0 ± 1.1	1.0 (0.0, 1.0)	0.0–5.0		
		Control	1.3 ± 1.2	1.0 (0.0, 2.0)	0.0–6.0		
	3^rd^ FU (3 months p-t)	CalSuli-4-02	0.6 ± 0.9	0.0 (0.0, 1.0)	0.0–4.0		
		Control	1.0 ± 1.2	1.0 (0.0, 2.0)	0.0–5.0		
	Termination (6 months p-t)	CalSuli-4-02	0.4 ± 0.9	0.0 (0.0, 1.0)	0.0–5.0		
		Control	0.8 ± 1.1	0.0 (0.0, 1.0)	0.0–5.0		

*CI* confidence interval, *FU* follow-up, *OR* odds ratio (i.e. the estimated odds of getting a lower total score in children treated with CalSuli-4-02 divided by the estimated odds of getting a lower total score in children treated with the comparator homeopathic product as obtained from Proportional odds model), *p-t* post-treatment, *SD* standard deviation, ITT intention-to-treat analysis

### Secondary outcome parameter: severity of individual complaints and symptoms

Improvement of individual complaints’ and symptoms’ severity was observed in both treatment groups during the course of the study (results not shown) and the number of children with absence of the respective complaint and symptom increased over the time of the study period (Tables 4 and 5). Thus it can be subsumed in the statement that at Termination Visit each respective individual complaint was absent in minimum 81 out of 99 (81.8 %) children in the CalSuli-4-02 and in minimum 69 out of 101 (68.3 %) children in the control group and that minimum 80 out of 99 (80.8 %) children in the CalSuli-4-02 group and a minimum of 73 out of 101 (72.3 %) children in the control group were free of symptoms. The individual complaint, “appetite disorder”, was found to be significantly improved in the CalSuli-4-02 group compared to the control group after 21 days of treatment, 3 and 6 months post-treatment (Table 4). Furthermore, significantly more children in the CalSuli-4-02 group reported absence of “child’s activities impairment” from Baseline to after 21 days treatment and Termination Visit (Table 5).

**Table 4:** Individual complaints

Individual complaints	Visit	Absence of complaint				*χ* ^2^-Test	
		CalSuli-4-02 *N* = 99		Control *N* = 101		(df) statistics	*p*
		N	%	N	%		
Fatigability	Baseline (day 0)	*42*	*42.4*	*41*	*40.6*	-	-
	2^nd^ FU (day 21)	*72*	*72.7*	*61*	*60.4*	(2) 4.140	0.1262
	3^rd^ FU (3 months p-t)	*68*	*68.7*	*64*	*63.4*	(2) 1.498	0.4729
	Termination (6 months p-t)	*81*	*81.8*	*73*	*72.3*	(1) 2.776	0.0957
Cough	Baseline (day 0)	*41*	*41.4*	*49*	*48.5*	-	**-**
	2^nd^ FU (day 21)	*84*	*84.9*	*84*	*83.2*	(2) 0.166	0.9203
	3^rd^ FU (3 months p-t)	*91*	*91.9*	*83*	*82.2*	(2) 5.015	0.0815
	Termination (6 months p-t)	*89*	*89.9*	*95*	*94.1*	(2) 2.576	0.2759
Nasal discharge (blocked/runny nose)	Baseline (day 0)	*37*	*37.4*	*37*	*36.6*	-	**-**
	2^nd^ FU (day 21)	*82*	*82.8*	*72*	*71.3*	(2) 4.130	0.1268
	3^rd^ FU (3 months p-t)	*82*	*82.8*	*80*	*79.2*	(2) 0.628	0.7305
	Termination (6 months p-t)	*88*	*88.9*	*85*	*84.2*	(2) 1.165	0.5585
Appetite disorder	Baseline (day 0)	*39*	*39.4*	*39*	*38.6*	-	**-**
	2^nd^ FU (day 21)	*75*	*75.8*	*58*	*57.4*	(1) 7.707	0.0055
	3^rd^ FU (3 months p-t)	*81*	*81.8*	*64*	*63.4*	(1) 9.052	0.0026
	Termination (6 months p-t)	*82*	*82.8*	*69*	*68.3*	(1) 6.099	0.0135
Irritability	Baseline (day 0)	*51*	*51.5*	*52*	*51.5*	-	**-**
	2^nd^ FU (day 21)	*78*	*78.8*	*70*	*69.3*	(1) 2.412	0.1204
	3^rd^ FU (3 months p-t)	*81*	*81.8*	*76*	*75.3*	(1) 1.395	0.2375
	Termination (6 months p-t)	*88*	*88.9*	*84*	*83.2*	(1) 1.573	0.2098

*Df* degree of freedom, *FU* follow-up, *p-t* post-treatment, *χ* ^*2*^ chi-square, ITT intention-to-treat analysis

**Table 5:** Individual symptoms

Individual symptoms	Visit	Absence of symptom				*χ* ^2^-Test	
		CalSuli-4-02 *N* = 99		Control *N* = 101		(df) statistics	*p*
		N	%	N	%		
Fever	Baseline (day 0)	*82*	*82.8*	*79*	*78.2*	-	-
	2^nd^ FU (day 21)	*94*	*95.0*	*94*	*93.1*	(1) 0.380	0.5377
	3^rd^ FU (3 months p-t)	*93*	*93.9*	*92*	*91.1*	(1) 0.803	0.3701
	Termination (6 months p-t)	*96*	*97.0*	*98*	*97.0*	(1) 0.000	0.9884
Nasal discharge (rhinorrhea/mucopurulent)	Baseline (day 0)	*42*	*42.4*	*46*	*45.5*	-	-
	2^nd^ FU (day 21)	*82*	*82.8*	*86*	*85.2*	(2) 0.218	0.8968
	3^rd^ FU (3 months p-t)	*84*	*84.9*	*86*	*85.2*	(1) 0.003	0.9554
	Termination (6 months p-t)	*90*	*90.9*	*92*	*91.1*	(2) 1.079	0.5832
Skin pallor	Baseline (day 0)	*42*	*42.4*	*37*	*36.6*	-	-
	2^nd^ FU (day 21)	*65*	*65.7*	*55*	*54.5*	(1) 2.660	0.1029
	3^rd^ FU (3 months p-t)	*74*	*74.8*	*66*	*65.4*	(1) 2.223	0.1360
	Termination (6 months p-t)	*80*	*80.8*	*73*	*72.3*	(1) 2.184	0.1395
Rales in lungs	Baseline (day 0)	*87*	*87.9*	*87*	*86.1*	-	-
	2^nd^ FU (day 21)	*97*	*98.0*	*98*	*97.0*	(2) 2.985	0.2248
	3^rd^ FU (3 months p-t)	*95*	*96.0*	*92*	*91.1*	(1) 2.806	0.0939
	Termination (6 months p-t)	*97*	*98.0*	*97*	*96.0*	(1) 1.980	0.1594
Restlessness for unknown reason	Baseline (day 0)	*77*	*77.8*	*85*	*84.2*	-	-
	2^nd^ FU (day 21)	*91*	*91.9*	*94*	*93.1*	(2) 1.362	0.5061
	3^rd^ FU (3 months p-t)	*97*	*98.0*	*97*	*96.0*	(1) 1.980	0.1594
	Termination (6 months p-t)	*94*	*95.0*	*97*	*96.0*	(2) 1.027	0.5985
Atopic dermatitis manifestations	Baseline (day 0)	*61*	*61.6*	*66*	*65.4*	-	-
	2^nd^ FU (day 21)	*73*	*73.7*	*72*	*71.3*	(2) 0.675	0.7137
	3^rd^ FU (3 months p-t)	*82*	*82.8*	*76*	*75.3*	(3) 3.208	0.3607
	Termination (6 months p-t)	*88*	*88.9*	*81*	*80.2*	(3) 5.070	0.1667
Child’s activities impairment	Baseline (day 0)	*63*	*63.6*	*71*	*70.3*	-	-
	2^nd^ FU (day 21)	*93*	*93.9*	*82*	*81.2*	(2) 8.218	0.0164
	3^rd^ FU (3 months p-t)	*92*	*92.9*	*87*	*86.1*	(1) 3.002	0.0832
	Termination (6 months p-t)	*95*	*96.0*	*87*	*86.1*	(1) 7.475	0.0063

*Df* degree of freedom, *FU* follow-up, *p-t* post-treatment, *χ* ^*2*^ chi-square, ITT intention-to-treat analysis

### Secondary outcome parameter: treatment satisfaction

**Figure 3:**
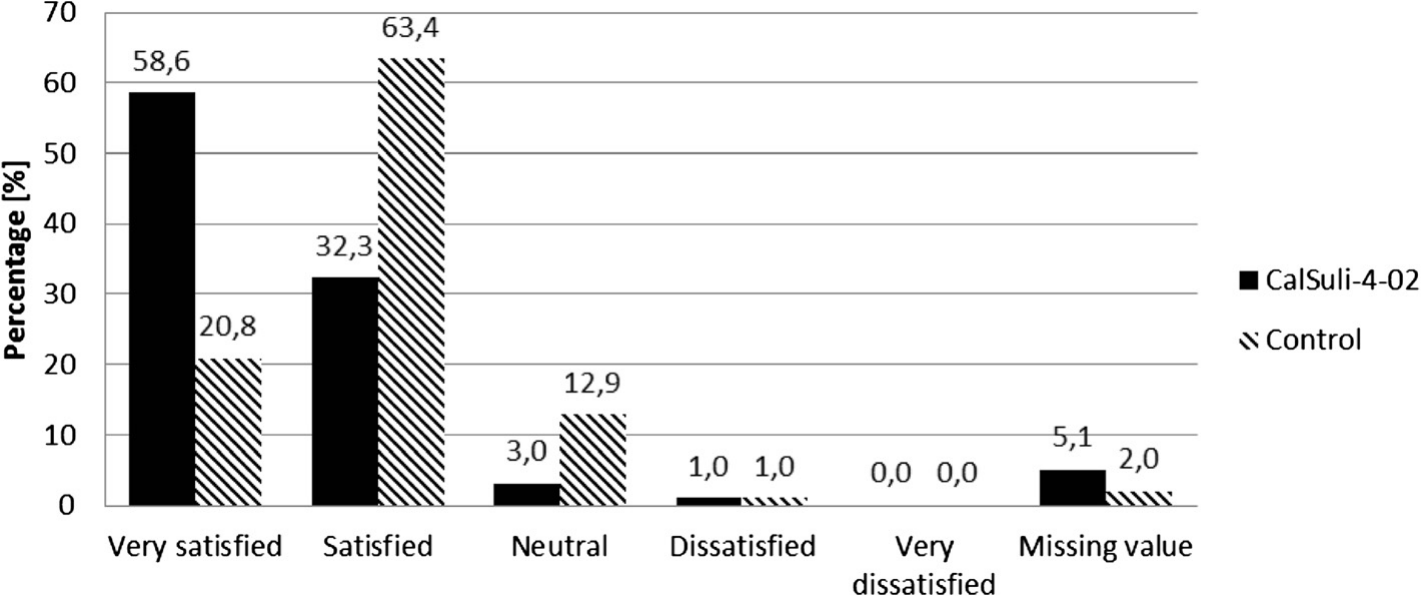
Treatment satisfaction assessment by children/parents by means of IMPSS at Termination Visit. *IMPSS* Integrative Medicine Patient Satisfaction Scale, ITT intention-to-treat analysis

As shown in Fig. 3, according to IMPSS assessment at the Termination Visit there were more children/parents in the CalSuli-4-02 group who were “very satisfied” (58 out of 99 (58.6 %)) with the treatment (*χ*^2^-Test: *p < 0.0001; ITT*) than in the control group (21 out of 101 (20.8 %)). At all other Visits, significant higher treatment satisfaction ratings for the CalSuli-4-02 group were found either (results not shown).

### Secondary outcome parameter: use of antibiotics

Table 6 shows that at baseline, the majority of children/parents in both treatment groups reported to use antibiotics within 12 months prior to study start either “sometimes” or “always” (CalSuli-4-02: 52 out of 99 (52.5 %); Control: 53 out of 101 (52.5 %)) when having an URTI or other ENT diseases. Despite some missing values, the percentages of children that had “sometimes” or “always” used antibiotics when they experienced an URTI or other ENT diseases were strongly decreased within the 1–3 months post-treatment assessed at 3^rd^ FU Visit (CalSuli-4-02: 9 out of 99 (9.1 %); Control: 20 out of 101 (19.8 %)) and the 4–6 months post-treatment assessed at Termination Visit (CalSuli-4-02: 7 out of 99 (7.1 %); Control: 17 out of 101 (16.8 %)). The decreased use of antibiotics was comparable between both treatment groups at 3^rd^ FU Visit (*χ*^2^-Test: *p = 0.0934; ITT*) and Termination Visit (*χ*^2^-Test: *p = 0.1274; ITT*).

**Table 6:** Change in the use of antibiotics

Antibiotics use for URTI and ENT diseases	Baseline (12 months prior to study start)				3^rd^ FU Visit (months 1-3 of post-treatment)				Termination Visit (months 4-6 of post-treatment)			
	CalSuli-4-02 group *N* = 99		Control group *N* = 101		CalSuli-4-02 group *N* = 99		Control group *N* = 101		CalSuli-4-02 group *N* = 99		Control group *N* = 101	
	N	%	N	%	N	%	N	%	N	%	N	%
Always	18	18.2	11	10.9	2	2.0	8	7.9	2	2.0	5	5.0
Sometimes	34	34.3	42	41.6	7	7.1	12	11.9	5	5.1	12	11.9
Seldom	27	27.3	35	34.7	14	14.1	12	11.9	8	8.1	4	4.0
Never	20	20.2	12	11.9	65	65.7	53	52.5	65	65.7	60	59.4
Missing	0	0.0	1	1.0	11	11.1	16	15.8	19	19.2	20	19.8

*FU* follow-up, ITT intention-to-treat analysis

### Secondary outcome parameter: safety assessment

In the CalSuli-4-02 group, 10 out of 99 (10.1 %) children reported a total of 11 AEs. Four of those AEs in three children were evaluated as ADRs (dermatitis atopic with moderate severity and probable causal relationship (2 x), hyperreflexia with moderate severity and probable causal relationship, bronchitis with mild severity and unlikely causal relationship). In the control group, 2 out of 101 (2.0 %) children reported a total of two AEs of which one was evaluated as an ADR (rhinorrhea with mild severity and unlikely causal relationship). Serious AEs didn’t occur in both treatment groups. One child in the CalSuli-4-02 group discontinued treatment and withdrew from the study due to the experience of two ADRs.

### Secondary outcome parameter: tolerability of treatment

**Fig. 4:**
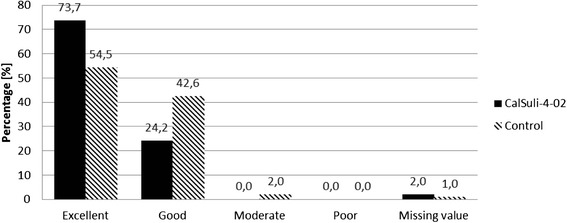
Assessment of study medications’ tolerability by investigator at the end of treatment period (day 21). Intention-to-treat analysis

Figure 4 shows that at the end of the treatment period (day 21) the tolerability of both homeopathic medicinal products was rated by the investigator mostly as “excellent” or “good” with significantly more often “excellent” ratings in the CalSuli-4-02 group (*χ*^2^-Test: *p < 0.05*; *ITT*). Comparable results were obtained for the 1^st^ FU and Termination Visit (data not shown). Children's/parents' assessment was in accordance with the investigator’s assessment but no significant difference between the groups was found on Termination Visit (*χ*^2^-Test: *p = 0.0712; ITT*).

## Discussion

Main finding of the present study was that 3 weeks treatment with CalSuli-4-02 and comparator complex homeopathic product resulted in comparable frequencies of acute URTIs, within 3 and 6 months post-treatment. However, CalSuli-4-02 was significantly more effective than the comparator in several other outcome parameters. First of all, CalSuli-4-02 demonstrated overall higher odds of having fewer complaints at the study end leading to a better overall health status. Specifically, it was observed that children’s appetite improved upon treatment with CalSuli-4-02. It has been reported that 60–72 % (depending on age) of children with acute URTIs suffer from appetite disorders or poor appetite, for as long as 17 days [20]. Since a poor appetite in children is a common parental worry [21], the improvement in the child’s appetite as observed with CalSuli-4-02 may thus be of great relevance for parents. Overall, higher odds of experiencing less URTI-related symptoms were also observed with CalSuli-4-02. Absence of child’s activities impairment was significantly more reported with CalSuli-4-02, indicating for example that children were able to be active and play. Not being able to play is a symptom that is also frequently observed in children with URTIs (up to 60 %), and may last up to 11 days with each experienced URTI [20]. Any improvement in the ability of their child to be active and play, may, therefore, lower the burden and distress in parents. Furthermore, satisfaction with treatment, as assessed by children/parents, was higher with CalSuli-4-02 compared to the comparator. High treatment satisfaction of children and parents with homeopathy are in line with studies previously published [10, 11, 22]. The mechanisms by which CalSuli-4-02 may exert its effect were not investigated in the present study and are currently unknown. A general principle in homeopathy is that a homeopathic medication can stimulate the body’s own adaptive healing processes. Through this stimulation the organism is enable to initiate a systemic self-reorganization toward more robust functioning as a whole [5]. Further studies are clearly necessary to investigate how CalSuli-4-02 may exert its effect on prevention of URTIs by reinforcing the child’s immune system along with decreasing the susceptibility to catch URTIs.

Both complex homeopathic medical treatments in the present study appeared to be safe, since a low percentage (1–3 %) of children reported ADRs which were mild or moderate in intensity. Similar low percentages of reported ADRs were observed in an outpatient clinical observational trial in Italy in which the occurrence of ADRs with homeopathic treatment were rare and not severe or serious [23] and in a study in which acute URTIs in children were also treated with a homeopathic complex product [24]. The tolerability of CalSuli-4-02 as assessed by investigators and children/parents was significantly better rated at the end of treatment period than the comparator homeopathic product.

The present study has its strengths and its limitations. A strength of the study was that children were randomized and that allocation of children to treatment was concealed until study start. Furthermore, a large number of children was included in the study and the drop-out rate was very low, increasing the precision of estimates. A limitation of the present study was that complaints and symptom severity (except of fever) were subjectively assessed by investigators or investigators and children/parents, using non-validated scales, and only gave information on the children’s health status at the time of the visit and not of the whole period since the last visit. Another limitation of this study was that the use of antipyretics and/or symptomatic medications (other than antibiotics) for treatment of symptoms of acute URTIs or ENT diseases were not monitored as part of the trial documentation. Since these medications were only allowed to treat acute symptoms of URTIs, and not for prophylactic reasons, it is unlikely that possible differences in concomitant mediations between the two groups had an effect on the primary outcome in the present study. However, a possible influence of these medications on secondary outcome parameters complaints and symptoms severity in both groups cannot be ruled out. Furthermore, the present study was of an open-label design as both, investigators and children/parents, knew which of the two treatments the children received. It can, therefore, not be excluded that the assessment of the more subjective outcome parameters such as symptom severity, treatment satisfaction, and tolerability were subject to expectation bias both for CalSuli-4-02 and the comparator, which was already registered for the prevention of acute URTIs. Another limitation of the current study design was that it had not included a third arm, a control group with children that received no treatment or a placebo. On average children experienced a median number of 6.0 acute URTIs in the 12 months period prior to study start (thereof at least three per child in the last 6 months) and ended with the occurrence of median 2.0 acute URTIs after 6 months post-treatment. Since the 6 months post-treatment period is shorter than the 12 months reference period before study start and seasonal influence might be considered, the assessment of the primary objective by means of Poisson regression modelling was primarily focused on treatment group comparison within this shorter period rather than evaluation of changes in the frequency of occurrences of acute URTIs in terms of a whole year. However, to test for possible seasonal influence, the variable was included as a covariate into the statistical model and it was shown that there was no statistically significant seasonal influence on the outcome and that the observed reduction in frequency of acute URTIs was comparable in both treatment groups. In a case series study by Ramchandani [17], individual homeopathic treatment was shown to prevent the occurrence of URTIs. In this study children suffered on average of 6.8 URTIs in a period of 6 months prior to treatment start, which decreased upon homeopathic treatment to 1.8 URTIs at 6 months FU. In two randomized placebo-controlled trials, the efficacy of single homeopathic medications was investigated for the prevention of URTIs in children [25, 26]. In these studies, no significant effect of homeopathy over placebo was found, possibly explained by the lack of effect of the homeopathic medications or by the process of selecting the right homeopathic medications. Very recently, a three-arm randomized clinical trial was published in which a complex and a single homeopathic medication were compared to placebo in the prevention of influenza and URTI in children [27]. One year post-intervention, treatment with both homeopathic medications led to a significant reduction of influenza and URTIs compared to placebo [27]. This study strengthens the findings of the present study in which the reduction in URTIs was also observed in both groups treated with a homeopathic medication.

An interesting finding of the current study was that both homeopathic treatments reduced the use of antibiotics. Antibiotics are frequently prescribed to treat URTIs, although it is often inappropriate due to the frequent viral origin of URTIs [3]. High and unnecessary antibiotic use is one of the main reasons for antibiotic resistances, which poses nowadays a growing threat to global public health [28, 29]. Therefore, there is a world-wide urgent need to develop and implement strategies that reduce inappropriate use of antibiotics [30]. Homeopathic treatment may be such an alternative treatment strategy to reduce the use of antibiotics [31]. A cohort study showed that patients with URTI, who visited a general practitioner that also practiced homeopathy, had lower consumption of antibiotics compared to patients visiting a physician who only practiced regular medicine [32]. Furthermore, in an open non-randomized observational study children with otitis media treated with individual homeopathic single medications used less antibiotics with better or comparable outcomes than those treated with regular medicine such as nasal drops, antibiotics, secretolytics and/or antipyretics [33]. Both for physicians as well as parents, homeopathic medications such as CalSuli-4-02 could, therefore, be an antibiotic sparing measure. In a review on complementary and alternative medicine therapies and antibiotic resistance, it was suggested that homeopathy could be recommended during the “watch and wait” period in acute otitis media and the time between testing and verifying pharyngitis’ potential bacterial origin, before prescribing antibiotics [34]. Recently, a Dutch consortium was initiated to further investigate the effectiveness of promising complementary and alternative medicine therapies as alternative therapeutic tools to control infectious diseases in humans [28].

## Conclusions

The present study demonstrated a comparable reduction of URTIs in both treatment groups. However, CalSuli-4-02 led to significantly less URTI-related complaints and symptoms and higher treatment satisfaction and tolerability. The observed reduction in antibiotics use upon treatment with the homeopathic medications under investigation, without the occurrence of complications, suggests that CalSuli-4-02 may be a promising treatment strategy to tackle antibiotic resistance. Further research is warranted to investigate the potential of CalSuli-4-02 as an antibiotic sparing option in children with recurrent URTIs.

## Abbreviations

ADRs: adverse drug reactions
AEs: adverse events
ANCOVA: analysis of covariance
CI: confidence interval
df: degree of freedom
ENT: ear, nose and throat
FU: follow-up
ICD: International statistical Classification of Diseases and related health problems
ICH: International Conference on Harmonization
IMPSS: Integrative Medicine Patient Satisfaction Scale
ITT: intention-to-treat
OR: odds ratio
POM: proportional odds model
p-t: post-treatment
RF: Russian Federation
RR: relative risk
SD: standard deviation
URTIs: upper respiratory tract infections
WHO: Word Health Organization
*χ*^2^-Test: chi-square-test
